# Intradural Extramedullary Cavernous Malformation of the Spinal Cord with Hemorrhagic Transformation and Rapid Expansion

**DOI:** 10.1155/2022/8677298

**Published:** 2022-08-12

**Authors:** Prabhat Poudel, Amrit K Chiluwal, Mohsen Nouri

**Affiliations:** ^1^Nepal Medical College Teaching Hospital, Kathmandu, Nepal; ^2^Jamaica Hospital Medical Center, Jamaica, Queens, NY, USA

## Abstract

Intradural extramedullary cavernous malformations in the spinal cord are rarely occurring vascular lesions. Mostly they are clinically silent unless the hemorrhagic transformation causes subarachnoid hemorrhage or neurologic deficits. We report the case of a 51-year-old man who developed a headache and weakness of the lower limb. Spinal cord magnetic resonance imaging revealed that the cause of his symptoms was a spinal intradural and extramedullary cavernous malformation with hemorrhagic transformation causing subarachnoid hemorrhage and compression of the thoracic spinal cord. Surgical decompression of the spinal cord followed by the resection of the lesion resulted in significant neurological improvement. Early diagnosis and early surgical extirpation of the lesion should be done to prevent recurrent hemorrhagic transformation and development of neurological symptoms.

## 1. Introduction

Cavernous angiomas of the nervous system are a collection of low-flow vascular malformations lined by thin leaky walls and devoid of smooth muscles and intervening neuronal or glial tissue [1]. Spinal cavernomas are extremely rare lesions with an overall incidence rate of 0.04–0.05% in populations [2]. In a population-based study of patients 50 to 89 years old undergoing MRI for nonclinical purposes, the prevalence of cavernous malformations was approximately 1 in 200 patients, but only 1 in 2700 had symptoms [3]. Most spinal cord cavernomas are clinically silent unless a hemorrhagic transformation causes mass effects, thus producing neurological symptoms [4].

In this paper, we report a case of subarachnoid hemorrhage and weakness of the limb due to intradural extramedullary cavernous malformation with rapid hemorrhagic transformation and expansion.

## 2. Case Presentation

A 51-year-old man with end-stage renal disease (ESRD) undergoing hemodialysis came to the emergency room (ER) from home with an acutely developed severe throbbing headache that started during the hemodialysis session earlier that day. The headache was localized in the posterior part of his head and shooting down his neck. He also complained of excruciating pain on his chest and his lower back; he initially denied any nausea or vomiting but began feeling nauseated and started retching while in the ER. The ER vital exam read his blood pressure to be 188/96 mm of Hg. The patient was alert and oriented and had no focal neurological deficits. On physical examination, the neck was rigid and flexion caused back pain to exacerbate. Complete neurological exam did not reveal any other abnormalities.

He underwent a CT angiogram of abdomen and pelvis with and without contrast to rule out abdominal aortic aneurysm and dissection which revealed no such findings. CT scan of the head failed to reveal any definite or large acute intracranial hemorrhage, and the evaluation for small subarachnoid hemorrhage was limited in the presence of the recently administered IV contrast and renal insufficiency contributing to dural venous and arterial enhancement. CT scan performed with and without contrast to rule out aneurysm or dissection of the thoracic aorta did not reveal such an aneurysm. Gadolinium could not be used because of the ESRD. On contrast-free brain magnetic resonance imaging, an abnormal signal was observed that extended along the clivus and into the visualized upper cervical spine that could be related to subarachnoid hemorrhage; however, a mass could not be ruled out. Three-D time-of-flight MRA of the head without contrast also revealed a 3 mm protrusion laterally along the cavernous part of the left internal carotid artery.

**Figure 1:**
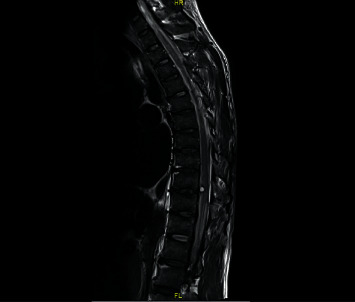
T2MRI of the entire spine showing an abnormal signal in the spinal canal and a hyperintensity of 5 mm in the anterior spinal canal likely intradural and extramedullary at the lower T9 level.

Magnetic resonance imaging of the entire spine without contrast (Figure 1) revealed more of the abnormal signal that extends from the clivus to the spinal canal, perhaps related to subarachnoid hemorrhage, but a mass could not be excluded without intravenous contrast. A hyperintensity with a surrounding rim of low signal on T2-weighted images measuring 5 mm in diameter was observed in the anterior spinal canal at the lower T9 level, likely to be intradural and extramedullary in location (Figure 1). The diagnostic spinal angiogram did not show any angiographically evident vascular malformations in the spinal cord.

**Figure 2:**
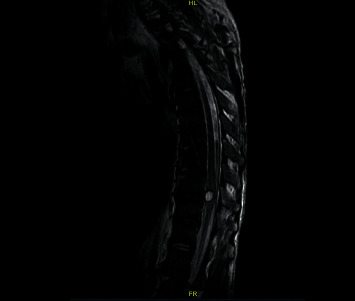
T2 MRI of the entire spine demonstrating a significant increase in the size of the previously noted lesion surrounded by a rim of a decreased gradient signal suggesting a hemorrhagic component and a likely picture of cord edema at T9-T10.

The patient acutely developed bilateral lower extremity weakness which was examined to be 2/5 on the left lower extremity and between 1/5 (proximal) and 2/5 (distal) on the right lower extremity. A thoracic cord MRI (Figure 2) demonstrated a lesion of 0.8 × 1.1 × 1.1 cm within the spinal canal at the level of T9 that appeared intradural and extramedullary in location and increased significantly in size compared to the previous study, and a surrounding rim of decreased gradient signal was noted suggesting a hemorrhagic component. There was also compression of the thoracic spinal cord at this level with a signal abnormality within the cord that spanned T9 to T10 and was compatible with associated cord edema. A diffuse hyperintense T1 signal outlining the thoracic spinal cord was noted to be compatible with hemorrhage within the thecal sac. A decision was made to surgically decompress the spinal cord and resect the lesion.

At the operation, baseline neuromonitoring showed the absence of motor evoked potential in the right lower extremity and a reduced signal on the left side. The spinal processes of T9, T10, and T11 were removed, bilateral laminectomy, partial facetectomy, and foraminotomy were performed, and when the dura was incised under a microscope, an organized clot emerged from the incision with high pressure. Organized subarachnoid hemorrhage could be seen on the dorsal aspect of the cord and spinal cord was pushed to the left side from the hemorrhage and the lesion. The entire tumor was removed en bloc, and it was measured to be 15 × 15 mm in size. Histopathological findings suggested cavernous angioma.

**Figure 3:**
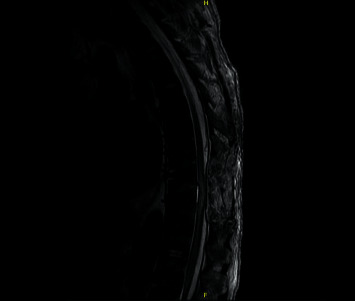
Postoperative MRI sagittal view showing T8–T10 laminectomy with mild dilatation of the cord and a T2 signal abnormality within the cord from the T7-8 through the T10-11 levels.

**Figure 4:**
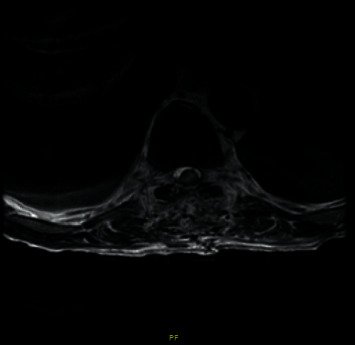
Postoperative MRI axial view showing the little fluid collected at the intradural and extramedullary space where the tumor was located.

Postoperative MRI (Figures 3 and 4) shows T8 to T10 laminectomy, a small fluid collection at the site of the operative bed, mildly dilated but not compressed cord, and a T2 signal abnormality within the cord from the T7-8 through the T10-11 levels. The patient is doing well, recovering neurologically, and is now being rehabilitated. He continues to have hemodialysis and has no new neurological complaints.

## 3. Discussion

Cavernous angiomas of the central nervous system are clinically silent, angiographically occult, and slow to grow and can expand by recurrent small bleeding and hemosiderin deposition, by the growth of cavernous nidus over time, and by acute massive hemorrhage causing acute mass effects [5].

There are sporadic and familial cases of cavernous angioma in the central nervous system. Cavernomas arise from the loss of an negative regulation of MEKK3-KLF2/4 signaling in endothelial cells, and endothelial TLR4 and the Gram negative microbiome in the gut are primary upstream stimulators of MEKK3-KLF2/4 [6]. Multiple cavernomas can also develop after exposure to radiation in radiotherapy or stereotactic radiosurgery [7].

Sporadic cavernoma lesions exist singly and often with a concomitant developmental venous anomaly, which is also considered responsible for their pathogenesis and natural history [8]. In the spinal intradural extramedullary space, cavernoma may arise from the blood vessels of the nerve roots in the cauda equina, the inner surface of the dura, or the pial surface of the spinal cord [9].

Hemorrhage and subsequent compression of neurological tissue from an intradural extramedullary cavernoma are the major reasons for the development of clinical symptoms. Although in the cavernoma, blood flows under very low pressure and therefore bleeding usually displaces without damaging the surrounding parenchyma, recurrent hemorrhagic transformation is a major issue that warrants definite management to prevent future neurological deficits. A systematic pooled analysis found that prior hemorrhage is a significant risk factor for hemorrhagic transformation of a cavernoma [10] and the most common causes of hemorrhagic transformation are the presence of other vascular malformations, coagulopathies, hypertension, trauma, and increased venous pressure.

Intradural extramedullary cavernous hemangioma is found primarily in men and occurs mainly at the thoracic level, and most of them are accompanied by back pain and radiculopathy with progressive sensory and motor deficits [11]. Significant bleeding can occur in more than a quarter of cases of cavernoma [12], and subarachnoid hemorrhage may also present as the initial symptom due to the high vascularity of the lesion [13]. Very rarely, acute hemorrhage may result in sudden onset of paraplegia [14].

MRI with standard sequences and susceptibility weighted imaging is the diagnostic test of choice [15]. The appearance of a reticulated core of mixed signal intensity and a surrounding rim of decreased signal intensity on T2-weighted MRI suggests the presence of cavernous angioma [16]. This appearance is likely due to the presence of mixed subacute and chronic hemorrhage in the lesion, evident by mixed components of high and low signal intensity and macrophages laden with hemosiderin in the periphery [17]. Spinal cavernomas are not identifiable by spinal angiography.

Only surgical removal of the lesion can improve symptoms and prevent disease progression. Moreover, worsening symptoms and acutely developing neurological deficits warrant immediate surgical response. Surgical extirpation of the symptomatic cavernoma lesion through a unilateral laminectomy approach within three months of initial presentation is a good treatment option with good long-term outcomes [18]; however, long-term stability may also be possible in conservatively treated oligosymptomatic patients [19].

## 4. Conclusions

We report a case of a 51-year-old man with spinal cord intradural extramedullary cavernous malformation who developed subarachnoid hemorrhage and weakness of the lower limb. MRI was useful to localize the lesion, and surgical removal of the lesion was done. The patient showed a significant neurological improvement and is now undergoing rehabilitation. Cavernous malformations of the spine can be asymptomatic for a long time and may be acutely symptomatic due to hemorrhage and subsequent expansion in size. Hemorrhage is more likely to occur in patients who have had hemorrhage from the lesions in the past. Surgical extirpation of the lesion is the best way to prevent recurrent hemorrhage and consequent neurological sequelae.
